# Preventive Medication Patterns in Bipolar Disorder and Their Relationship With Comorbid Substance Use Disorders in a Cross-National Observational Study

**DOI:** 10.3389/fpsyt.2022.813256

**Published:** 2022-05-03

**Authors:** Romain Icick, Ingrid Melle, Bruno Etain, Margrethe Collier Høegh, Sébastien Gard, Sofie R. Aminoff, Marion Leboyer, Ole A. Andreassen, Raoul Belzeaux, Chantal Henry, Thomas D. Bjella, Jean-Pierre Kahn, Nils Eiel Steen, Frank Bellivier, Trine Vik Lagerberg

**Affiliations:** ^1^Norwegian Centre for Mental Disorders Research (NORMENT), Division of Mental Health and Addiction, Oslo University Hospital, Institute of Clinical Medicine, University of Oslo, Oslo, Norway; ^2^FondaMental Foundation, Créteil, France; ^3^INSERM U1144, Université Paris Cité, Paris, France; ^4^Institute of Clinical Medicine, University of Oslo, Oslo, Norway; ^5^Université Paris Cité, Paris, France; ^6^Assistance Publique – Hôpitaux de Paris, GH Saint-Louis – Lariboisière – F. Widal, Département de Psychiatrie, Paris, France; ^7^Hôpital Charles Perrens, Centre Expert Trouble Bipolaire, Pôle de Psychiatrie Générale et Universitaire (3/4/7), Bordeaux, France; ^8^Early Intervention in Psychosis Advisory Unit for South East Norway, Division of Mental Health and Addiction, Oslo University Hospital, Oslo, Norway; ^9^Paris Est Créteil, INSERM U955, IMRB, Laboratoire Neuro-Psychiatrie Translationnelle, Créteil, France; ^10^Assistance Publique – Hôpitaux de Paris (AP-HP), HU Henri Mondor, Département Medico-Universitaire de Psychiatrie et d’Addictologie (DMU ADAPT), Fédération Hospitalo-Universitaire de Médecine de Precision (FHU IMPACT), Créteil, France; ^11^Assistance Publique – Hôpitaux de Marseille (AP-HM), Hôpital Sainte-Marguerite, Pôle de Psychiatrie, INT-UMR 7289, CNRS, Aix-Marseille University, Marseille, France; ^12^Department of Psychiatry, Service Hospitalo-Universitaire, GHU Paris Psychiatrie & Neurosciences, Paris, France; ^13^Université de Lorraine, CHRU de Nancy et Pôle de Psychiatrie et Psychologie Clinique, Centre Psychothérapique de Nancy, Laxou, France

**Keywords:** bipolar disorder, substance use disorder, treatment guidelines, tobacco smoking, comorbidity

## Abstract

**Objective:**

The potential role of sub-optimal pharmacological treatment in the poorer outcomes observed in bipolar disorder (BD) with vs. without comorbid substance use disorders (SUDs) is not known. Thus, we investigated whether patients with BD and comorbid SUD had different medication regimens than those with BD alone, in samples from France and Norway, focusing on compliance to international guidelines.

**Methods:**

Seven hundred and seventy patients from France and Norway with reliably ascertained BD I or II (68% BD-I) were included. Medication information was obtained from patients and hospital records, and preventive treatment was categorized according to compliance to guidelines. We used Bayesian and regression analyses to investigate associations between SUD comorbidity and medication. In the Norwegian subsample, we also investigated association with lack of medication.

**Results:**

Comorbid SUDs were as follows: current tobacco smoking, 26%, alcohol use disorder (AUD), 16%; cannabis use disorder (CUD), 10%; other SUDs, 5%. Compliance to guidelines for preventive medication was lacking in 8%, partial in 44%, and complete in 48% of the sample. Compliance to guidelines was not different in BD with and without SUD comorbidity, as was supported by Bayesian analyses (highest Bayes Factor = 0.16). Cross national differences in treatment regimens led us to conduct country-specific adjusted regression analyses, showing that (1) CUD was associated with increased antipsychotics use in France (OR = 2.4, 95% CI = 1.4–3.9, *p* = 0.001), (2) current tobacco smoking was associated with increased anti-epileptics use in Norway (OR = 4.4, 95% CI = 1.9–11, *p* < 0.001), and (3) AUD was associated with decreased likelihood of being medicated in Norway (OR = 1.2, 95% CI = 1.04–1.3, *p* = 0.038).

**Conclusion:**

SUD comorbidity in BD was overall not associated with different pharmacological treatment in our sample, and not related to the level of compliance to guidelines. We found country-specific associations between comorbid SUDs and specific medications that warrant further studies.

## Introduction

Bipolar disorder (BD) is a chronic and relapsing condition associated with a high burden for individuals, caregivers, and societies (1). This burden is strongly associated with the high level of comorbidity in BD (2, 3). Comorbid substance use disorders (SUDs, including nicotine dependence/tobacco smoking) are found in up to 50–60% individuals with BD (4–6). Compared to BD alone, the presence of comorbid SUD (BD + SUD) has been associated with poorer outcomes, including premature mortality (7), higher rates of suicide attempts (8), and suicide mortality (9), as well as delayed remission from acute mood episodes (10). The presence of comorbid SUDs may complicate the pharmaceutical management of BD (11); e.g., tobacco use disorders have been associated both with a more severe psychopathology, as shown by our group (8) and others (12), and complicated pharmaceutical management (13). Beyond age and gender, additional dimensions related to abnormal self-awareness might contribute to increased SUD risk in BD, namely sensation seeking (14) and anxiety (15). These may co-exist in individuals with particularly complex BD course in case of, e.g., comorbid borderline personality disorders (16), further increasing the likelihood of complicated pharmaceutical management.

To date, there is no specific guideline for the pharmaceutical treatment of BD + SUD (17). Indeed, guidelines are often limited by the fact that they are typically based on the results of randomized controlled double-blind trials, which include selected BD patients. Consequently, patients with psychiatric comorbidities such as SUDs are often excluded. Moreover, a substantial proportion of BD patients show inadequate response to medication (18). Medication patterns in community BD samples and naturalistic settings often diverge from guidelines, increasing the risk of poor clinical outcome (19). This includes scarce lithium use (20), polypharmacy (21), frequent antidepressant (22), and benzodiazepine use (23) despite lack of evidence for their efficacy in BD and additional risk of addiction for the latter (24).

Comorbid SUDs are may play a role in both the lack of treatment response and the use of non-recommended medication regimens in BD for several reasons. Firstly, psychoactive substances can elicit a wide range of BD symptoms [e.g., psychotic and manic symptoms with cannabis (25)], which may increase the need for symptomatic treatment. Secondly, substance use also alters the pharmacodynamics [e.g., amphetamines (26)] and the pharmacokinetics [e.g., tobacco and P450 enzymes (13)] of medications for BD. Thirdly, BD + SUD has been associated with reduced treatment adherence compared to BD alone (27) – although this may be accounted for by impulsiveness (28). Fourthly, both clinicians’ and patients’ perceptions might influence prescription attitudes and modify the pharmaceutical treatment of BD in case of comorbid SUD. This might be due to lower psychoeducation level, increased stigma, or lack of confidence in treatment efficacy (29, 30). With that regards, one study reported no difference of medication profiles in BD + SUD vs. BD inpatients at discharge (31). Two other studies, although not specifically aimed at comparing BD with vs. without SUDs, reported discrepant results. One study conducted among homeless persons with BD showed that comorbid SUDs were significantly associated with inappropriate prescription regimens (32), while a nationwide French cohort study (independent from the sample analyzed in the current study) did not observe any difference in preventive BD medication in outpatients with vs. without SUDs (33). Given the paucity of available literature, knowledge about the sources of variability (34) and non-compliance to guidelines of pharmacological treatment in BD + SUD remains limited. Furthermore, the clinical management of BD patients can be affected by local customs, expert opinions, and differences in treatment availability. Likewise, the epidemiology of SUD also shows major cross-national differences. This warrants cross-national comparisons to disentangle the effects of SUDs from national trends in SUD and medication usage.

To investigate this issue, we used data from a large, well-characterized sample of patients with BD from France and Norway. Our objective was to investigate whether the presence of SUDs would be associated with different preventive medication regimens, including more frequent deviations from European guidelines, differences in the use of individual medication classes, and different likelihood of receiving current preventive medication. We further aimed to clarify whether putative relationships between medication regimens and SUDs are independent from clinical and demographic variables, especially country of inclusion.

## Materials and Methods

This was a *post hoc* study of a sample of patients with ascertained BD recruited in France (2000–2012) and Norway (2003–2020). Both original studies aimed to extensively characterize BD in order to inform future prevention and treatment strategies, using similar assessment protocols.

### Participants

Inclusion criteria for France were: (1) age ≥18 years; (2) meeting criteria for a diagnosis of BD-I or BD-II disorder according to the Diagnostic and Statistical Manual for Mental Disorders, 4th edition, text revised (DSM-IV-TR) (35); and (3) willingness and ability to provide written informed consent. In France, participants also had to (1) be under preventive medication and be euthymic at inclusion, as defined by a Montgomery-Asberg Depression Rating Scale (MADRS) score ≤8 (36) and a Young Mania Rating Scale (YMRS) score of ≤5 (37); (2) master the French language. Moreover, in France, ability to provide written informed consent also required the absence of clinically significant cognitive impairment, which was assessed using clinical judgment. In Norway, although euthymia was not a formal inclusion criterion, participants had to be clinically stable and to master a Scandinavian language. Also, specific effort was made to include cases early in their first treatment for BD. Additional exclusion criteria in Norway were: (1) history of severe head trauma and (2) intellectual disability. For Norwegian cases, who participated in a neurocognitive assessment we used an estimated IQ based on two subtests of the WAIS with a good concordance with total IQ. For a small subset of participants who did not attend the neurocognitive assessment, we undertook a comprehensive review of educational attainment, school grades, and general interview performance to rule out the presence of intellectual disability (which is defined as an IQ < 70).

Written informed consent was obtained from all participating patients in both countries. In France, The Research Ethics Board of Pitié-Salpêtrière Hospital reviewed and approved this study. In Norway, the project was approved by the Regional Committee for Medical Research Ethics and the Norwegian Data Inspectorate. This involved being registered in the database and having one’s data analyzed for research purposes.

### Study Sample

**FIGURE 1:**
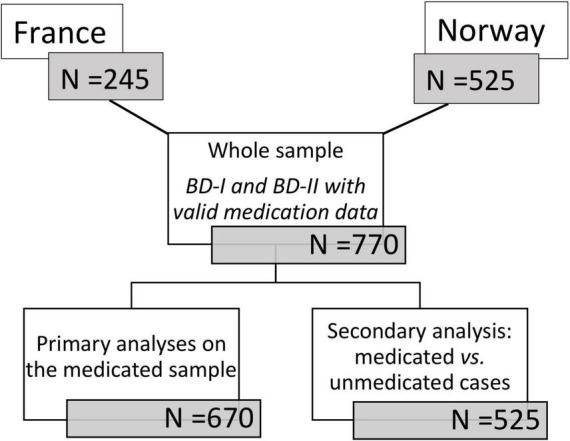
Flowchart for sample selection.

A total of 770 patients with BD-I (*n* = 526) or BD-II (*n* = 244) and reliable medication status were included. Recruitment was consecutive in both countries. Patients who sought treatment for BD in psychiatric units were evaluated for eligibility for study participation by their treating clinician. We do not know how many who refused to participate, but of those referred, the refusal rate was <5%. Due to ethical regulation, data about patients, who refused to enter the study could not be analyzed. The study of treatment compliance to guidelines and individual medication classes was performed in 670 medicated cases from France and Norway. All French cases received some medication at the time of inclusion in line with inclusion criteria. They were therefore excluded from the medicated vs. unmedicated analysis. Thus, the comparison of medicated vs. unmedicated status was performed in 525 cases from Norway only (Figure 1).

### Clinical Assessment

Trained psychiatrists, medical doctors, and clinical psychologists carried out clinical assessments aimed at providing reliable lifetime DSM-IV BD and SUD diagnoses in both samples. Investigators used the Diagnostic Interview for Genetic Studies [DIGS (38)] in France and the Structured Clinical Interview for DSM-IV axis-I disorders [SCID-I (39)] in Norway. The course of BD was also extensively characterized.

### Substance Use Assessments

Tobacco smoking was defined as smoking on a daily basis – a reliable proxy of DSM-IV nicotine dependence (40). In the French subsample, lifetime tobacco smoking (former + current) was assessed, while in the Norwegian subsample, only current tobacco smoking was considered. As such, tobacco smokers in the French subsample (*N* = 160) were both current (*N* = 99) and former smokers (*N* = 61), whereas those from the Norwegian subsample were current smokers only (*N* = 261). Diagnoses of abuse or dependence to other substances were combined to obtain single binary variables of “use disorder” for alcohol and cannabis use disorders (hereafter termed AUD and CUD, respectively), yielding the following categories: current tobacco smoking, lifetime AUD, lifetime CUD, and lifetime SUDs not related to tobacco nor alcohol nor cannabis, hereafter termed “other SUDs.” Additionally, we kept the possibility of analyzing all SUDs that were not AUD, i.e., CUD + “other SUDs,” in case the subgroups would be deemed too small and/or yielded borderline associations.

### Medication Regimens

In both countries, current medications were recorded and categorized by the investigator into: lithium, anti-epileptics (valproate derivatives including valpromide, carbamazepine, lamotrigine), antipsychotics, antidepressants, and benzodiazepines. The sample can be considered as naturalistic with regards to medications since participants were recruited with their treatment as prescribed by the clinician in charge, which was thus unrelated to the current study (although being medicated was an inclusion criterion in the French sample).

#### Treatment Compliance to Guidelines

Firstly, we categorized the sample in relation to level of compliance to recommendations for preventive treatment of international guidelines [e.g., NICE (41), CANMAT/ISBD (42)], where lithium, several antiepileptics (valproate/valpromide/carbamazepine/lamotrigine) and antipsychotics are considered first-line mood-stabilizers. Antiepileptics and antipsychotics with primary indication in BD were identified from the Norwegian and French national recommendations. Compliance to those guidelines was deemed absent when the participant was using antidepressant or benzodiazepine without mood-stabilizer, partial if any antidepressant or benzodiazepine was used together with mood-stabilizer and complete when no antidepressant or benzodiazepines and any mood-stabilizer was used. Importantly, we focused on preventive treatment, since the samples are euthymic or next-to-euthymic and the range of episode-specific treatments was deemed too large. Regardless of underlying mood-stabilizing treatment, we considered that antidepressants and benzodiazepines remained not fully compliant in the maintenance phase of BD. Such medications are often used at some point in the course of BD, whether during the initial – often undiagnosed – phase where unspecific depressive and anxiety can prevail (43), to alleviate symptoms of comorbid anxiety disorders (3), or for the acute treatment of depressive episodes. Benzodiazepines and/or antidepressants are not easily discontinued. This may be due to prevasive residual symptoms (44) and/or clinicians’ beliefs and patients’ anticipatory anxiety regarding medication cessation (28). However, they have been associated with a wide range of adverse features in BD, including manic symptoms and rapid cycling for antidepressants (45) and cognitive impairment and addictive disorders for benzodiazepines (23). Additionally, both the possible causes and consequences of prescribing antidepressants and/or benzodiazepines in BD have been associated with SUD comorbidity in BD (46, 47), further warranting the focus on these medication classes as proposed in the current study.

#### Individual Medical Classes

Secondly, we analyzed each individual medication class and their relationship to SUD and key sociodemographic and clinical variables, by country.

#### Medicated vs. Unmedicated

Thirdly, in the Norwegian subsample we were also able to compare SUD rates in those not using any psychotropic treatment (“unmedicated”) vs. those receiving psychotropic medication (“medicated”). Here, we excluded cases in their first treatment episode for (hypo)mania (*n* = 195), as preventive treatment may not yet have been initiated in these cases. We analyzed the “medicated” status separately because we anticipated that this would be associated with different patient histories and clinical correlates as compared to guidelines compliance and medication regimens. In order to explore these results further, we also present data from a subsample of 161 cases, who filled in both the *Medication Adherence Rating Scale* (MARS) (48) to measure adherence, and the *Beliefs about Medicines Questionnaire* (BMQ) (49) to measure the general attitude toward medicine and medication and to estimate how much the patients’ concerns overcome his/her perceived needs for medication, using the general and the specific subscales. Of note, these secondary analyses are provided for discussion purposes only.

### Statistics

Data are described as means (standard deviation, SD), medians (interquartile range, IQR) or counts (frequency). Bivariate tests were performed for SUDs only and medication-related variables, namely: in the sample as a whole and – if any of these variables exhibited cross-national differences – in each country, separately for compliance to guidelines and individual medication classes (lithium, anti-epileptics, antipsychotics, antidepressants, and benzodiazepines) and in the Norwegian subsample for the status “being medicated.” We used trend tests for variables with >2 groups and Chi-squared or Fisher’s exact tests for the others, based on a threshold for statistical significance at *p* < 0.05 (two-tailed tests). In order to verify the null hypothesis when a lack of difference in the medication pattern according to the SUD status will be observed, we computed Bayes factors (BF) with the R package *BayesFactor*. A BF can take any decimal value above zero. A value of 1 indicates equal evidence for both the H_1_ and H_0_ hypotheses. The more the value closes to zero, the stronger evidence for an absence of difference. To interpret BFs, we used the recommended thresholds (50) (Supplementary Table 1).

Each medication pattern variable (compliance to guidelines, specific medication classes and being medicated vs. unmedicated) significantly associated with one of the SUD variables was used as the dependent variable into regression models to ascertain the independence of associations from potential confounders. These confounders were chosen when they were associated with a given medication variable, at *p* < 0.05, two-tailed bivariate tests. In the case of a lack of association between and SUDs and our main medication-related variables – namely: compliance to guidelines and the status of “being medicated,” an exploratory regression model was still performed in order to fully test our main hypotheses. All analyses were conducted with R version 4.0.2 (51) through R studio version 1.3.1093 for Mac OS^®^ X.14.6. A summary of the packages that were used is available as a Supplementary Methods.

## Results

### Description of Medication and Substance Use Disorder in the Whole Sample (*n* = 670)

Compliance with international guidelines was distributed as follows: absent in 53 (8%) cases, partial in 296 (44%) cases, and complete in the remaining 321 (48%) cases. A majority of patients (55%) reported polypharmacy. Current smoking was reported by 174 participants (26%). AUD was diagnosed in 104 (16%), CUD in 66 (10%), and other SUDs in 28 (5%) patients (Table 1).

**TABLE 1:** Description of the medicated sample, as a whole, and by country.

	Whole medicated sample	*N*	Norway	France	Test value	*p*-Value Norway vs. France
	*N* = 670		*N* = 425	*N* = 245		
Gender (women vs. men)	402 (60%)	670				
Age***	36 (27–47)	670				
Site (Norway vs. France)	425 (63%)	670				
BD-II subtype (vs. BD-I)	190 (28%)	670				
AAO of BD*	21.0 (17–28)	528				
BD duration***	13.0 (7–23)	528				
Rate of MDE/year of BD***	0.3 (0.1–0.8)	480				
Rate of (hypo)manic episodes/year of BD*	0.4 (0.1–1.4)	363				
History of psychosis	394 (60%)	669				
Lifetime SA**	205 (39%)	525				
Current tobacco smoking***	174 (26%)	670				
Lifetime AUD*	104 (16%)	662				
Lifetime CUD	66 (10%)	664				
Other SUD lifetime*	28 (5%)	523				
Compliance to treatment guidelines						
Complete	321 (48%)	670	221 (52%)	100 (41%)		0.006
Partial	296 (44%)		168 (40%)	128 (52%)	10.2	
Absent	53 (8%)		36 (9%)	17 (7%)		
Current lithium treatment***	196 (30%)	661	99 (42%)	97 (23%)	25.7	<0.001
Current anti-epileptic treatment***	256 (38%)	670	97 (23%)	99 (42%)	14.3	<0.001
Current antipsychotics treatment***	329 (49%)	669	139 (33%)	117 (48%)	51.1	<0.001
Current antidepressant treatment***	289 (43%)	669	254 (60%)	75 (31%)	0.0215	0.883
Current benzodiazepine treatment***	124 (19%)	790	185 (44%)	104 (43%)	60.3	<0.001

Data are given as N (%) or median (IQR). Significant association with compliance to treatment guidelines in the whole sample are marked as *p < 0.05, **p < 0.01, ***p < 0.001. Tests and p-values are from Chi-squared, Fisher’s, or Mann–Whitney tests for differences between Norway and France, uncorrected.

*BD, bipolar disorder; AAO, age at onset; MDE, major depressive episode; SA, suicide attempt; AUD, alcohol use disorder; CUD, cannabis use disorder; SUD, substance use disorder.*

### Compliance to Guidelines Across Substance Use Disorders

We found no difference in terms of compliance to guidelines regarding comorbid SUDs (Table 2); fully consistent with Bayes Factors (Supplementary Figure 1), which indicated strong evidence for a lack of difference. In ordinal logistic regression, neither current smoking, AUD or CUD were associated with non-guideline compliant treatment (lowest *p*-value = 0.21 for CUD). However, in this model, female gender (OR = 1.6, *p* = 0.014) and BD-II subtype (OR = 2.6, *p* < 0.001) remained independently associated with lower compliance to guidelines (data not shown).

**TABLE 2:** Variables associated with compliance to treatment guidelines in the whole medicated sample (*N* = 670).

Compliance with international guidelines	Complete	Partial	Absent	Test value	*p*-Value	*N*
	*N* = 321	*N* = 296	*N* = 53			
Gender (women vs. men)*	176 (55%)	189 (64%)	37 (70%)	7.53	0.023	670
Age*	34 (26–45)	39 (28–48)	33 (28–49)	6.51	0.039	670
Site (Norway vs. France)**	221 (69%)	168 (57%)	36 (68%)	10.2	0.006	670
BD-II subtype (vs. BD-I)***	59 (18%)	103 (35%)	28 (53%)	37.4	<0.001	670
AAO of BD	22 (18–30)	20 (17–28)	20 (15–27)	2.666	0.264	528
BD duration	11 (6–22)	14 (8–23)	13 (6–28)	4.851	0.088	528
Rate of MDE/year of BD**	0 (0–1)	0 (0–1)	0 (0–1)	11.751	0.003	479
Rate of (hypo)manic episodes/year of BD	0 (0–1)	0 (0–2)	0 (0–2)	5.6811	0.125	363
History of psychosis***	218 (69%)	154 (53%)	22 (42%)	22.9	<0.001	660
Lifetime SA**	72 (30%)	118 (47%)	15 (41%)	14.2	0.001	525
Current tobacco smoking	78 (24%)	86 (29%)	10 (19%)	3.32	0.19	670
Lifetime AUD	46 (14%)	50 (17%)	8 (15%)	0.93	0.628	662
Lifetime CUD	28 (9%)	31 (11%)	7 (13%)	1.3	0.521	664
Other SUD lifetime*	10 (4%)	17 (7%)	1 (3%)	NA*^a^*	0.456	523

Data are given as N (%) or median (IQR). *p < 0.05, **p < 0.01, ***p < 0.001. Tests and p-values are from Chi-squared, Fisher’s, or Kruskal–Wallis tests.

*BD, bipolar disorder; AAO, age at onset; MDE, major depressive episode; SA, suicide attempt; AUD, alcohol use disorder; CUD, cannabis use disorder; SUD, substance use disorder. Other SUDs refer to SUDs not related to alcohol, nor cannabis.*

*^a^Fisher’s exact test.*

### Individual Medication Classes Across Substance Use Disorders

There was no significant difference in individual medication classes as a function of SUDs (Supplementary Table 2), which was supported by Bayes Factors as well (Supplementary Figure 1). The complete medication patterns as a function of SUD comorbidity is shown in Supplementary Figure 2. Since there were significant differences in the proportion of French vs. Norwegian cases regarding compliance to guidelines (Table 1) and every individual medication classes but antidepressants (highest *p* = 0.006), we further characterized country effects and country-specific medication regimens.

Norwegian cases were more likely than the French to receive compliant treatment (52 vs. 41%, overall *p* = 0.006), probably due to the higher proportion of French cases receiving treatment with partial compliance to guidelines (40 vs. 52%). This was likely driven by large differences in benzodiazepine use (10 vs. 34%). Additionally, the absence of compliance to guidelines seemed more frequent in Norway compared to France (9 vs. 7%), which further legitimated country-specific follow-up analyses of the relationship between (1) SUDs and compliance to guidelines and (2) SUDs and individual medication classes, as shown below.

### Country-Specific Associations Between Substance Use Disorders and Compliance to Guidelines

Both BFs (Supplementary Figure 2) and exploratory ordinal regressions (data not shown) supported an absence of country effect in the compliance to guidelines (lowest *p*-values = 0.21 for AUD in France and 0.45 for CUD in Norway, respectively).

### Country-Specific Associations Between Substance Use Disorders and Individual Medication Classes

**FIGURE 2:**
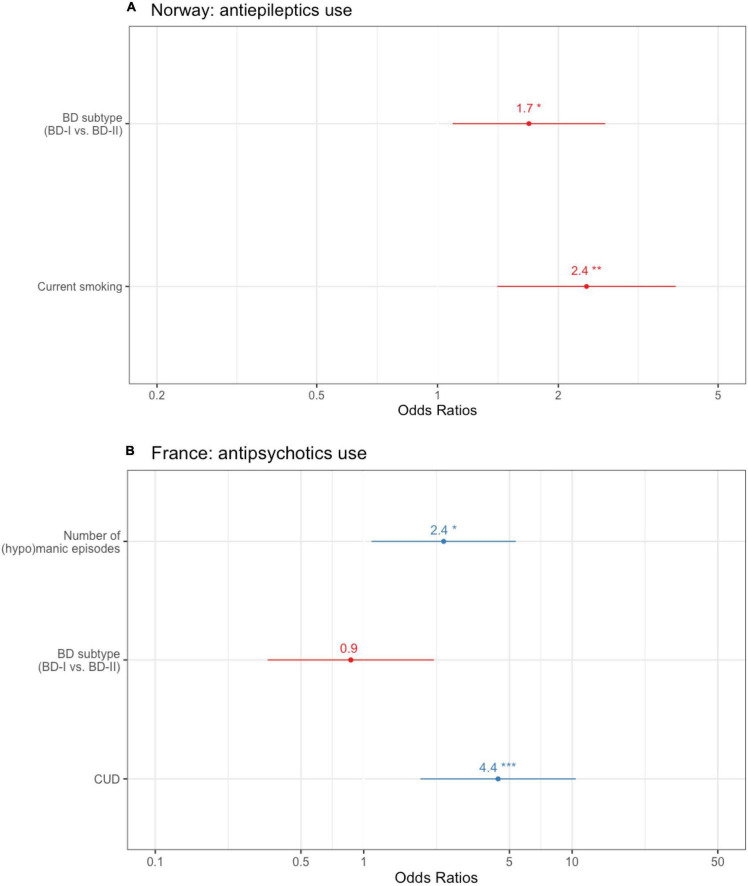
Country-specific binary logistic regressions with **(A)** anti-epileptics use in the Norwegian subsample (*N* = 425) and **(B)** antipsychotics use as the dependent variable in the French subsample (*N* = 243; the SUD predictor of interest is CUD). Bar length indicates 95% confidence interval. AAO, age at onset; BD, bipolar disorder; CUD, cannabis use disorder. Other SUD refers to SUDs not related to alcohol, nor cannabis. *p < 0.05, **p < 0.01, ***p < 0.001.

In Norway (Supplementary Table 3), antiepileptics use was more frequent in current compared to former + never smokers (*p* = 0.001). Follow-up binary regressions showed that tobacco smoking remained significantly associated with increased antiepileptics use (OR = 2.4, 95% CI = 1.4–3.9, *p* = 0.001) after controlling for the effects of BD subtype (BD-II vs. BD-I, OR = 1.7, 95% CI = 1.1–2.6, *p* = 0.019) (Figure 2A). The AUC of the model was 0.68, based on 239 cases. There was no other association between individual SUD and individual medication classes in the Norwegian subsample.

In France (Supplementary Table 4), antipsychotics use was more frequent in case of lifetime CUD (*p* < 0.001). This was confirmed by binary regression, where CUD remained significantly associated with antipsychotics use (OR = 4.4, 95% CI = 1.9–11, *p* < 0.001) after controlling for the effect of BD subtype (*p* = 0.8), and history of psychosis (OR = 2.2, 95% CI = 1.1–5.6, *p* = 0.03) (Figure 2B). The AUC of the model was 0.77 based on 191 cases. There was no other association between individual SUDs and individual medication classes in the French subsample.

### Substance Use Disorder and Medicated vs. Unmedicated Cases

The Norwegian subsample comprised 274 (83%) medicated and 56 (17%) unmedicated cases after exclusion of first-treatment cases (*n* = 195). Being medicated vs. unmedicated had no significant association with any SUD (Table 3).

**TABLE 3:** Variables associated with the current medicated status in the Norwegian subsample, who was not in their first mood episode.

	Unmedicated	Medicated	Test value	*p*-Value	Effect size (95% CI)	*N*
	*N* = 56	*N* = 274				
Gender (women vs. men)	33 (59%)	166 (61%)	0.0065	0.936	1.1 (0.6, 1.9)	330
Age	34 (24–46)	36 (27, 46)	6973	0.282	−0.13 (−0.45, 0.17)	330
BD-II subtype (vs. BD-I)**	33 (59%)	97 (35%)	9.82	0.002	2.6 (1.5, 4.7)	330
AAO of BD*	18 (14–22)	20 (16.8–27)	2778	0.016	−0.43 (−0.81, −0.09)	237
BD duration	15 (8–26)	12.0 (7–20)	4277	0.132	0.28 (−0.09, 0.66)	237
Lifetime SA	6 (17%)	66 (33%)	3.11	0.078	2.4 (1.0–6.8)	236
History of psychosis	26 (46%)	154 (57%)	1.78	0.182	1.5 (0.9–2.8)	325
Rate of MDE/year of BD	0.4 (0.1–1)	0.4 (0.2–0.9)	2878	0.895	0.09 (−0.32, 0.49)	219
Rate of (hypo)manic episodes/year of BD**	1 (0.2–3)	0.4 (0.2–1)	4816	0.004	0.4 (0.07–0.75)	237
Current tobacco smoking	4 (7%)	50 (18%)	3.42	0.065	2.8 (1.1–9.8)	330
Lifetime AUD	11 (20%)	33 (12%)	1.71	0.191	0.6 (0.3–1.2)	330
Lifetime CUD	8 (14%)	20 (7%)	NA*^a^*	0.111	0.5 (0.2–1.2)	330
Other SUDs	4 (11%)	13 (6%)	NA*^a^*	0.308	0.6 (0.2–2.1)	240

Data are given as N (%) or median (IQR). *p < 0.05, **p < 0.01. Tests and p-values are from Chi-squared, Fisher’s, or Mann–Whitney tests. Effect size expressed as univariate odds ratio for categorical variable and Cohen’s d for continuous variables. Medicated status represents the reference group.

*BD, bipolar disorder; AAO, age at onset; MDE, major depressive episode; SA, suicide attempt; AUD, alcohol use disorder; CUD, cannabis use disorder; SUD, substance use disorder. Other SUDs refer to SUDs not related to alcohol, nor cannabis.*

*^a^Fisher’s exact test.*

**FIGURE 3:**
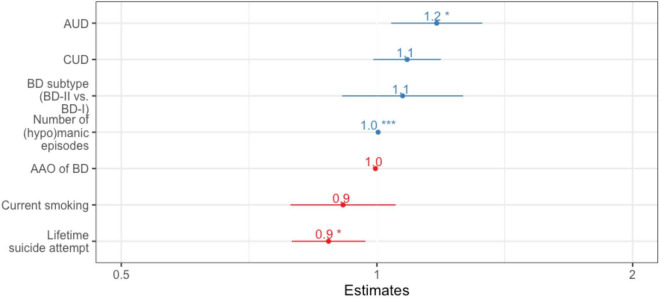
Forest plot for binary logistic regression in Norway, with “being unmedicated vs. medicated” as the dependent variable. *N* = 195 after exclusion of first-episode cases. Bar length indicates 95% confidence interval. AAO, age at onset; BD, bipolar disorder; AUD, alcohol use disorder; CUD, cannabis use disorder. *p < 0.05, **p < 0.01, ***p < 0.001.

When including current smoking and both lifetime AUD and CUD in a binary regression analysis (Figure 3), we uncovered an independent association between being unmedicated and AUD (OR = 1.2, 95% CI = 1.04–1.3, *p* = 0.038). Being currently unmedicated was also independently associated with a higher number of (hypo)manic episodes (OR = 1, 95% CI = 1.02–1.07, *p* < 0.001) and a lower probability of lifetime suicide attempt (OR = 0.88, 95% CI = 0.79–0.97, *p* = 0.014). AUC of this model was 0.78, based on 195 cases. Of note, we entered AAO of BD and the absolute number of (hypo)manic episodes together instead of the rate of (hypo)manic episodes in order to avoid multicollinearity and to be able to dissect the effects from both AAO and the number of episodes.

Finally, there was no indication that BD cases with comorbid SUD had higher resistance (lowest *p*-values = 0.499 for the BMQ-general and 0.374 for the BMQ-specific) or lower adherence (*p*-value = 0.39 for MARS) regarding their medication, as compared to BD cases without any SUD. Interestingly though, the BMQ necessity subscore was higher in the BD+AUD than in the BD alone group, *p* = 0.037.

## Discussion

In this study of a large sample of patients from France and Norway, who were extensively characterized for both BD and SUD history, we found no significant association between the compliance to pharmacological treatment guidelines and comorbid SUDs. Thus, our results suggest that it is feasible to follow existing guidelines to treat BD, also for patients with comorbid SUD. In line with this, no SUD was associated with individual medication classes in the sample as a whole. However, country-specific analyses identified independent associations between current tobacco smoking and anti-epileptics use and between AUD and being unmedicated in the Norwegian subsample; as well as between CUD and antipsychotics use in France. To the best of our knowledge, this study reports among the most detailed characterization of the links between comorbid SUD and preventive medication in BD, with a focus on both medication patterns and level of medication compliance to guidelines. Our main finding, which is negative, was ascertained with the computation of Bayes factors, meaning that we had adequate statistical power and that this finding can be considered as reliable. Importantly as regards generalizability, the medication regimens of our samples were similar to previous studies. For instance, in 7,406 individuals with BD-I, II and NOS diagnoses from the United States community (52), 18% would have been categorized as having non-compliant preventive treatment, 51% received polypharmacy, 24% benzodiazepines, and 71% antidepressants (the only category that seemed to differ from our sample). As for the prevalence of SUD, our sample remains within the range of tertiary care samples for BD (53, 54), which often show relatively low rates of SUDs compared to other clinical samples (5).

Available literature examining the possibility that comorbid SUD would be associated with non-evidence based treatment in BD reported either less specific or borderline findings, as compared to ours. One study found that BD-SUD inpatients showed less use of mood-stabilizers at discharge, as compared to BD only patients (31). The second study reported the absence of association between SUD and a reduced adherence to BD medication guidelines, but with *p* = 0.06 (55). This may be due to the smaller size of these samples. In a larger registry study (52), BD subjects with AUD or other SUDs showed a decrease in mood-stabilizers use during follow-up, as measured by medication possession ratio. Although this was primarily interpreted as lower adherence to treatment, the authors acknowledged that their measurement captured all kinds of treatment interruption. Thus, this finding was in line with that of Norwegian cases having less likelihood of proper preventive treatment for BD in case of comorbid AUD. Interesting as well in this study was that bipolar illness complexity was also associated with reduced mood-stabilizer use. More precisely, we replicated an association between reduced compliance to guidelines and BD-II vs. BD-I subtype (55), and evidenced an independent association between female gender and lack of compliance to guidelines, which had not been specifically reported previously (55). This finding was not due to common characteristics of BD associated with female gender (56, 57), most of which were controlled for in our study. However, this could have been due to other factors associated with antidepressant prescription, which was significantly higher in women vs. men (Supplementary Table 2) and likely drove the association between gender and compliance to guidelines in our study. This includes anxiety/anxiety disorders (58) and fear of weight gain (59). We suggest that the fear of weight gain could be much higher for mood-stabilizers and antipyschotics than for antidepressants, thereby increasing the likelihood for prescribing antidepressants as opposed to mood-stabilizers in women. In line with this, we found previous associations between female gender and complex polypharmacy in BD (60). Overall, these data highlight the need for further research regarding gender issues in patients’ and prescribers’ adherence to guidelines.

We investigated the correlates of being unmedicated in the Norwegian subsample. The regression analysis showed that comorbid AUD was associated with current lack of pharmacological treatment. AUD may increase the likelihood of delayed diagnosis/underdiagnosis of BD in these patients, especially if AUD preceded BD (61). Conversely, cocaine use disorders have been associated with a risk of overdiagnosing and/or precipitating BD (62, 63). Compared to BD without AUD, comorbid AUD in BD is rather associated with depressive symptoms in BD, including a positive correlation between depressive symptoms and alcohol craving (64), and – possibly – a more frequent depressive predominant polarity (65, 66). This may hamper identification of the BD and thus delay treatment. However, studies reporting associations between AUD and bipolar depression have often yielded discrepant results (67, 68), noting that merely all SUDs may predict longer time to recovery from bipolar depression (10, 67). In line with underdiagnosis, our results also raise the possibility that clinicians are less inclined to initiate mood-stabilizers in cases with continuous alcohol use, even in the presence of mood episodes. Thus, until the years 2010s, it was usually recommended to start such treatment after alcohol detoxification or – at least – after a large reduction in alcohol use (61). In line with this general hypothesis of difficult diagnosis/treatment choice in BD with vs. without AUD, we found no evidence of decreased adherence or increased concern/necessity ratio across AUD groups. This suggests that non-prescription may have prevailed over non-adherence regarding the unmedicated status associated with AUD in our sample. One of the key issues might be the consideration of current vs. past AUD (10) and of moderate vs. heavy alcohol drinking (69), the latter being more strongly associated with incident bipolar depression than the former (70).

In the Norwegian subsample, we also found an independent association between current smoking vs. past- and never-smoking and increased anti-epileptics use. We can hardly think of the rationale for this association. Anti-epileptics were also more commonly prescribed to BD-II cases, but this did not alter the association with current tobacco smoking. Other possible reasons due to gender differences (valproate being avoided in women of childbearing age) or to the clinical expression of BD were ruled out, yet, there may be some bias due to the fact that “non-current smokers” were a mixed group of never + former smokers. We did not retrieve previous evidence of such association in the literature, so that a pilot, prospective study on this specific issue with detailed data regarding the reasons for prescribing/choosing to take anti-epileptics seems warranted.

Cannabis use disorder has overall been associated with a heavy burden in BD (71, 72). In the French subsample, it was associated with increased use of antipsychotics, suggesting that clinicians may have needed to maintain these medications to manage persistent mood instability and/or psychotic symptoms.

### Limitations

The study was cross-sectional and medication data were collected by self-report, thus sensitive to recall bias and making us less able to disentangle non-prescription from patients’ non-adherence. We did not collect individual treatment names or dosages to assess fine-grained compliance to guidelines and polypharmacy. No correction was applied for multiple testing, however, we believe that using Bayes and regression analyses reduced the risk of both false positives and false negatives. We did not assess further comorbidity such as anxiety, personality and attention deficit/hyperactivity (ADHD) disorders, which have been associated with BD+SUD comorbidity (3) and could lead to altered medication regimens. We relied on lifetime SUD diagnoses, although the amount and recency of exposure to addictive substances may have played an additional role in prescription patterns, especially by encouraging clinicians to wait for abstinence before prescribing proper BD medication. Importantly, the associations evidenced here are likely bi-directional, without any possible conclusion about causal inference.

## Conclusion

Overall, SUDs were not associated with lack of compliance toward guidelines for preventive BD treatment in a large, cross-national sample. However, individuals with comorbid AUD were significantly less likely to be medicated in the Norwegian sample. Specific guidelines are lacking for the subgroup of BD+SUD cases, and treating clinicians in our study seem to have remained compliant to general guidelines for BD despite the presence of comorbid SUD. In the absence of specific treatment, available evidence thus suggests that intensive and early mood-stabilizing therapy can be used for BD+SUD. With that regards, more specific psychosocial treatments showed promise for BD+SUD cases (73, 74). We believe our study also highlights the fact that, in general, it is necessary to examine SUD comorbidity by individualizing tobacco, alcohol, cannabis, and other substances of abuse given that each of these categories showed relevant associations that would not have been uncovered if we had regrouped them. Moreover, our findings contribute to a better knowledge for both patients and clinicians. In dually diagnosed BD patients, integrated care and improved diagnostic and therapeutic strategies are urgently required. Some of these strategies have already shown promising results (46, 73, 75–77) and should be implemented in both psychiatric and addiction care settings.

## Data Availability Statement

The original contributions presented in the study are included in the article/Supplementary Material, further inquiries can be directed to the corresponding author/s.

## Ethics Statement

The studies involving human participants were reviewed and approved by the Comité de Protection des Personnes, Hôpital Pitié-Salpêtrière, and Regional Committee for Medical Research Ethics and Norwegian Data Inspectorate. The patients/participants provided their written informed consent to participate in this study.

## Author Contributions

RI, TL, and IM wrote the first draft of the manuscript. BE, MH, SG, SA, ML, OA, RB, CH, TB, J-PK, NS, and FB designed the initial study and recruited the sample. All authors have contributed to and critically reviewed the manuscript.

## Conflict of Interest

The authors declare that the research was conducted in the absence of any commercial or financial relationships that could be construed as a potential conflict of interest.

## Publisher’s Note

All claims expressed in this article are solely those of the authors and do not necessarily represent those of their affiliated organizations, or those of the publisher, the editors and the reviewers. Any product that may be evaluated in this article, or claim that may be made by its manufacturer, is not guaranteed or endorsed by the publisher.
