# Supplementary figures and images for: Preventive Medication Patterns in Bipolar Disorder and Their Relationship With Comorbid Substance Use Disorders in a Cross-National Observational Study
Source: Front Psychiatry. 2022 May 3;13:813256. doi: 10.3389/fpsyt.2022.813256 (PMC9110763; doi:10.3389/fpsyt.2022.813256)

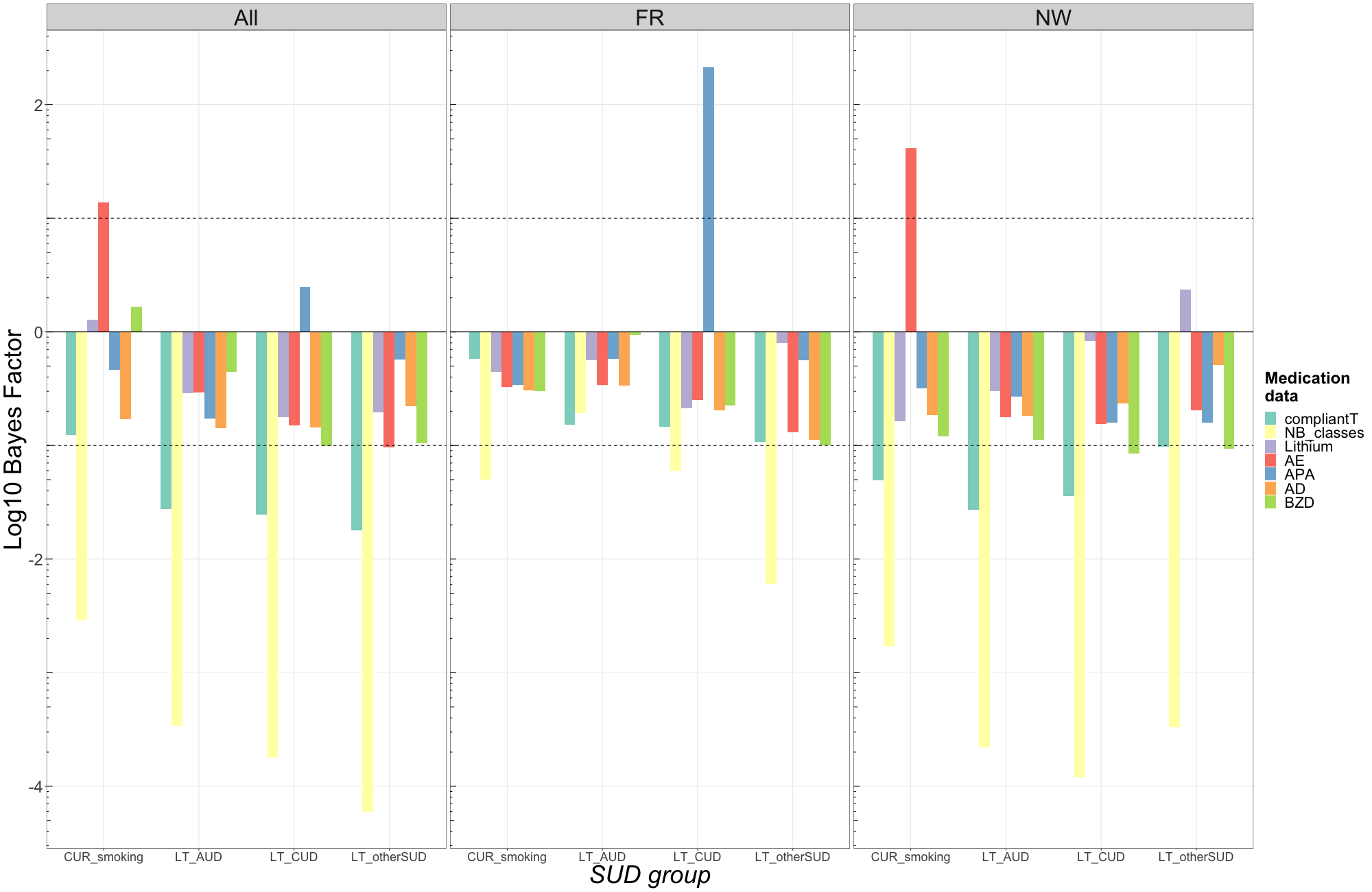

Supplement: Supplementary file 1 [file Image_1.PNG]

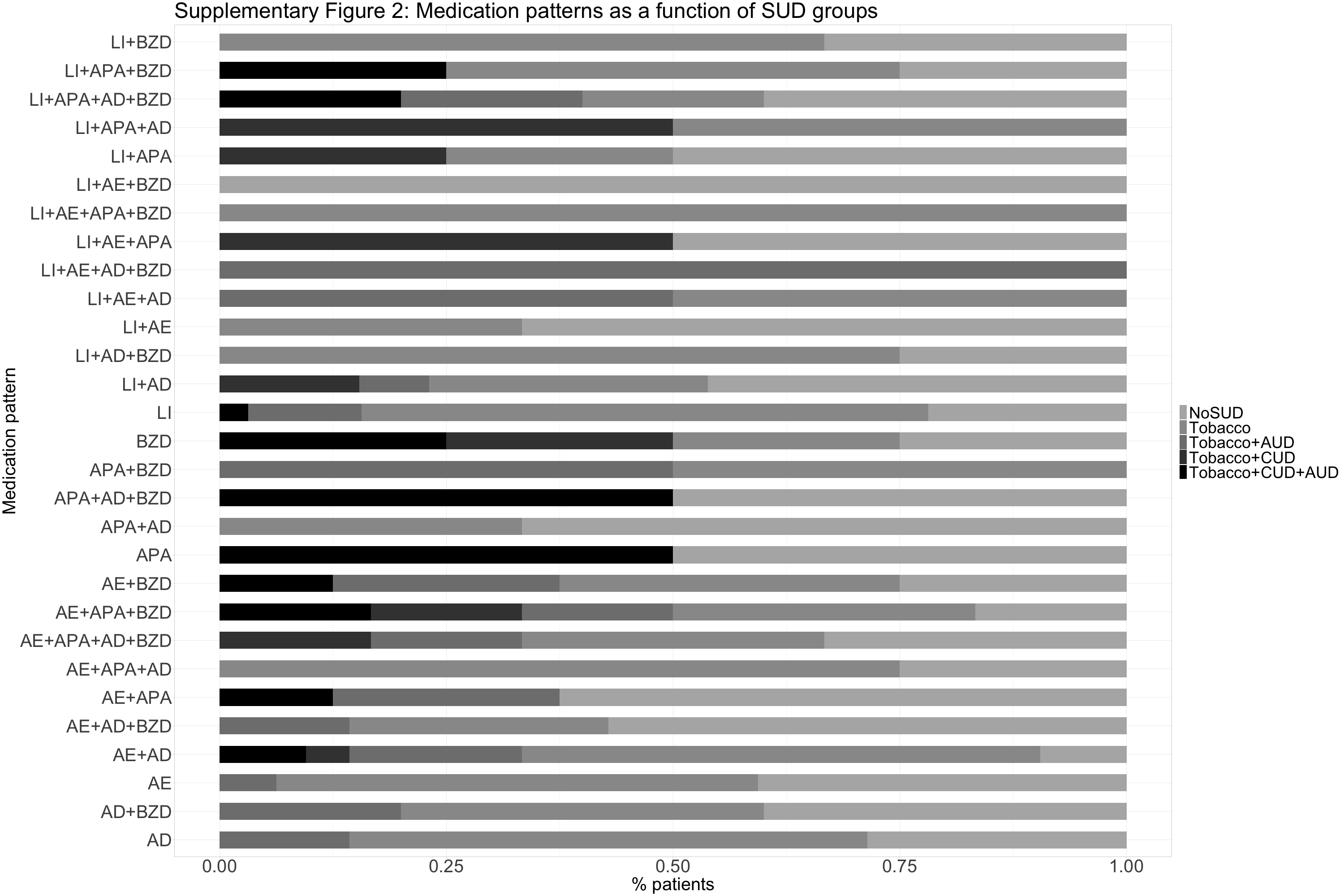

Supplement: Supplementary file 2 [file Image_2.PNG]
