# Supplementary material for: Preventive Medication Patterns in Bipolar Disorder and Their Relationship With Comorbid Substance Use Disorders in a Cross-National Observational Study
Source: Front Psychiatry. 2022 May 3;13:813256. doi: 10.3389/fpsyt.2022.813256 (PMC9110763; doi:10.3389/fpsyt.2022.813256)
Supplement: Supplementary file 3 [file Data_Sheet_1.DOCX]

R version 4.1.0 (2021-05-18)

Platform: x86_64-apple-darwin17.0 (64-bit)

Running under: macOS Mojave 10.14.6

Matrix products: default

BLAS: /System/Library/Frameworks/Accelerate.framework/Versions/A/Frameworks/vecLib.framework/Versions/A/libBLAS.dylib

LAPACK: /Library/Frameworks/R.framework/Versions/4.1/Resources/lib/libRlapack.dylib

locale:

[1] en_US.UTF-8/en_US.UTF-8/en_US.UTF-8/C/en_US.UTF-8/en_US.UTF-8

attached base packages:

[1] stats graphics grDevices utils datasets methods base

other attached packages:

[1] RcmdrMisc_2.7-2 sandwich_3.0-1 car_3.0-11 carData_3.0-4 cluster_2.1.2 pvclust_2.2-0

[7] forcats_0.5.1 stringr_1.4.0 dplyr_1.0.7 purrr_0.3.4 readr_2.0.2 tibble_3.1.5

[13] tidyverse_1.3.1 tidyr_1.1.4 scales_1.1.1 BayesFactor_0.9.12-4.2 Matrix_1.3-4 coda_0.19-4

[19] flextable_0.6.9 compareGroups_4.5.1 rstatix_0.7.0 ggpubr_0.4.0 ggplot2_3.3.5

loaded via a namespace (and not attached):

[1] utf8_1.2.2 tidyselect_1.1.1 lme4_1.1-27.1 htmlwidgets_1.5.4 grid_4.1.0 munsell_0.5.0

[7] codetools_0.2-18 effectsize_0.5 chron_2.3-56 withr_2.4.2 colorspace_2.0-2 highr_0.9

[13] knitr_1.36 uuid_0.1-4 rstudioapi_0.13 stats4_4.1.0 DescTools_0.99.43 ggsignif_0.6.3

[19] rcompanion_2.4.1 officer_0.4.0 labeling_0.4.2 emmeans_1.7.0 farver_2.1.0 datawizard_0.2.1

[25] vctrs_0.3.8 generics_0.1.0 TH.data_1.1-0 xfun_0.26 R6_2.5.1 assertthat_0.2.1

[31] multcomp_1.4-17 nnet_7.3-16 rootSolve_1.8.2.3 gtable_0.3.0 multcompView_0.1-8 lmom_2.8

[37] rlang_0.4.11 MatrixModels_0.5-0 systemfonts_1.0.3.9000 sjPlot_2.8.9 splines_4.1.0 broom_0.7.9

[43] rapportools_1.0 checkmate_2.0.0 yaml_2.2.1 reshape2_1.4.4 abind_1.4-5 modelr_0.1.8

[49] backports_1.2.1 HardyWeinberg_1.7.2 Hmisc_4.6-0 tools_4.1.0 tcltk_4.1.0 ellipsis_0.3.2

[55] kableExtra_1.3.4 jquerylib_0.1.4 RColorBrewer_1.1-2 Rsolnp_1.16 proxy_0.4-26 Rcpp_1.0.7

[61] plyr_1.8.6 base64enc_0.1-3 rpart_4.1-15 pbapply_1.5-0 summarytools_1.0.0 zoo_1.8-9

[67] haven_2.4.3 fs_1.5.0 magrittr_2.0.1 data.table_1.14.2 magick_2.7.3 openxlsx_4.2.4

[73] lmtest_0.9-38 reprex_2.0.1 truncnorm_1.0-8 mvtnorm_1.1-3 sjmisc_2.8.7 matrixStats_0.61.0

[79] hms_1.1.1 evaluate_0.14 xtable_1.8-4 rio_0.5.27 sjstats_0.18.1 jpeg_0.1-9

[85] readxl_1.3.1 gridExtra_2.3 ggeffects_1.1.1 compiler_4.1.0 mice_3.13.0 writexl_1.4.0

[91] crayon_1.4.1 minqa_1.2.4 htmltools_0.5.2 tzdb_0.1.2 Formula_1.2-4 libcoin_1.0-9

[97] expm_0.999-6 Exact_3.0 lubridate_1.8.0 DBI_1.1.1 sjlabelled_1.1.8 dbplyr_2.1.1

[103] MASS_7.3-54 boot_1.3-28 cli_3.0.1 pryr_0.1.5 parallel_4.1.0 insight_0.14.5

[109] pkgconfig_2.0.3 coin_1.4-2 foreign_0.8-81 xml2_1.3.2 svglite_2.0.0 bslib_0.3.1

[115] webshot_0.5.2 estimability_1.3 rvest_1.0.2 digest_0.6.28 parameters_0.14.0 rmarkdown_2.11

[121] cellranger_1.1.0 htmlTable_2.3.0 nortest_1.0-4 gld_2.6.2 gdtools_0.2.3 curl_4.3.2

[127] gtools_3.9.2 modeltools_0.2-23 nloptr_1.2.2.2 lifecycle_1.0.1 nlme_3.1-153 jsonlite_1.7.2

[133] viridisLite_0.4.0 fansi_0.5.0 pillar_1.6.3 lattice_0.20-45 fastmap_1.1.0 httr_1.4.2

[139] survival_3.2-13 glue_1.4.2 bayestestR_0.11.0 zip_2.2.0 png_0.1-7 pander_0.6.4

[145] class_7.3-19 stringi_1.7.5 sass_0.4.0 performance_0.8.0 latticeExtra_0.6-29 e1071_1.7-9
