# Supplementary material for: Preventive Medication Patterns in Bipolar Disorder and Their Relationship With Comorbid Substance Use Disorders in a Cross-National Observational Study
Source: Front Psychiatry. 2022 May 3;13:813256. doi: 10.3389/fpsyt.2022.813256 (PMC9110763; doi:10.3389/fpsyt.2022.813256)
Supplement: Supplementary file 4 [file Table_1.docx]

**Supplementary Table 1: interpretation of Bayes Factor**

| **BF** | **Level of evidence** |
| --- | --- |
| > 100 | Extreme evidence for H_1_ |
| 30 – 100 | Very strong evidence for H_1_ |
| 10 – 30 | Strong evidence for H_1_ |
| 3 – 10 | Moderate evidence for H_1_ |
| 1 – 3 | Anecdotal evidence for H_1_ |
| 1 | Equal evidence for H_1_ and H_0_ |
| 1/3 – 1 | Anecdotal evidence for H_0_ |
| 1/3 – 1/10 | Moderate evidence for H_0_ |
| 1/10 – 1/30 | Strong evidence for H_0_ |
| 1/30 – 1/100 | Very strong evidence for H_0_ |
| < 1/100 | Extreme evidence for H_0_ |

**Supplementary Table 2: SUDs and medication variables in the sample as a whole**

**Supplementary Table 3: Medication variables in the Norwegian subsample**

**Supplementary Table 4: Medication variables in the French subsample**
