# Supplementary material for: Preventive Medication Patterns in Bipolar Disorder and Their Relationship With Comorbid Substance Use Disorders in a Cross-National Observational Study
Source: Front Psychiatry. 2022 May 3;13:813256. doi: 10.3389/fpsyt.2022.813256 (PMC9110763; doi:10.3389/fpsyt.2022.813256)
Supplement: Supplementary file 5 [file Table_2.pdf]

Descriptive statistics and noinal associations of medication variables

	compliance					lithium				
	compliant	partly_compliant	non_compliant	p.trend	N	0	1	OR	p.overall	N
	N=321	*N=296*	*N=53*			*N=465*	*N=196*			
age	34 (26-45)	39 (28-48)	33 (28-49)	0.052	670	34 (27-46)	39 (29-49)	1.0 [1.0;1.0]	0.004	661
gender_txt:				0.006	670				0.948	661
Men	145 (45%)	107 (36%)	16 (30%)			188 (40%)	78 (40%)	Ref.		
Women	176 (55%)	189 (64%)	37 (70%)			277 (60%)	118 (60%)	1.0 [0.7;1.4]		
site:				0.057	670				<0.001	661
NW	221 (69%)	168 (57%)	36 (68%)			328 (71%)	97 (49%)	Ref.		
FR	100 (31%)	128 (43%)	17 (32%)			137 (29%)	99 (51%)	2.4 [1.7;3.4]		
bipolar_type:				<0.001	670				<0.001	661
1	262 (82%)	193 (65%)	25 (47%)			314 (68%)	164 (84%)	Ref.		
2	59 (18%)	103 (35%)	28 (53%)			151 (32%)	32 (16%)	0.4 [0.3;0.6]		
bipolar_duration	11 (6-22)	14 (8-23)	13 (6-28)	0.029	528	12 (6-23)	13 (8-23)	1.0 [1.0;1.0]	0.299	519
bipolar_AAO	22 (18-30)	20 (17-28)	20 (15-27)	0.111	528	21 (17-28)	22 (18-31)	1.0 [1.0;1.0]	0.210	519
Bipolar_any_psychotic: 1	218 (69%)	154 (53%)	22 (42%)	<0.001	660	273 (59%)	119 (62%)	1.1 [0.8;1.6]	0.539	651
MDE_year	0 (0-1)	0 (0-1)	0 (0-1)	0.001	479	0 (0-1)	0 (0-1)	1.1 [0.9;1.3]	0.818	473
UP_year	0 (0-1)	0 (0-2)	0 (0-2)	0.023	528	0 (0-1)	0 (0-1)	1.0 [0.9;1.1]	0.707	519
sa_ever: 1	72 (30%)	118 (47%)	15 (41%)	0.002	525	122 (35%)	77 (46%)	1.6 [1.1;2.3]	0.019	516
current_smoking: 1	78 (24%)	86 (29%)	10 (19%)	0.823	670	109 (23%)	61 (31%)	1.5 [1.0;2.1]	0.049	661
Misuse_alcohol_lifetime: 1	46 (14%)	50 (17%)	8 (15%)	0.523	662	78 (17%)	25 (13%)	0.7 [0.4;1.2]	0.245	653
Misuse_cannabis_lifetime: 1	28 (9%)	31 (11%)	7 (13%)	0.256	664	50 (11%)	16 (8%)	0.7 [0.4;1.3]	0.371	655
Misuse_cocaine_lifetime: 1	2 (1%)	4 (2%)	0 (0%)	0.852	532	4 (1%)	2 (1%)	1.1 [0.1;5.9]	1.000	523
Misuse_other_lifetime_bis: 1	10 (4%)	17 (7%)	1 (3%)	0.588	523	22 (6%)	6 (4%)	0.6 [0.2;1.4]	0.291	514

	antiepileptics					antipsychotics				
	0	1	OR	p.overall	N	0	1	OR	p.overall	N
	N=414	*N=256*				*N=340*	*N=329*			
age	35 (27-48)	38 (28-47)	1.0 [1.0;1.0]	0.134	670	38 (28-49)	34 (26-45)	1.0 [1.0;1.0]	0.002	669
gender_txt:				0.408	670				0.508	669
Men	160 (39%)	108 (42%)	Ref.			131 (39%)	136 (41%)	Ref.		
Women	254 (61%)	148 (58%)	0.9 [0.6;1.2]			209 (61%)	193 (59%)	0.9 [0.7;1.2]		
site:				<0.001	670				<0.001	669
NW	286 (69%)	139 (54%)	Ref.			171 (50%)	254 (77%)	Ref.		
FR	128 (31%)	117 (46%)	1.9 [1.4;2.6]			169 (50%)	75 (23%)	0.3 [0.2;0.4]		
bipolar_type:				0.116	670				<0.001	669
1	306 (74%)	174 (68%)	Ref.			213 (63%)	266 (81%)	Ref.		
2	108 (26%)	82 (32%)	1.3 [0.9;1.9]			127 (37%)	63 (19%)	0.4 [0.3;0.6]		
bipolar_duration	12 (6-21)	14 (8-24)	1.0 [1.0;1.0]	0.072	528	14 (8-25)	11 (6-21)	1.0 [1.0;1.0]	0.005	527
bipolar_AAO	21 (17-29)	21 (17-28)	1.0 [1.0;1.0]	0.976	528	21 (17-28)	22 (17-28)	1.0 [1.0;1.0]	0.838	527
Bipolar_any_psychotic: 1	248 (61%)	146 (57%)	0.8 [0.6;1.2]	0.351	660	157 (47%)	236 (73%)	3.1 [2.3;4.4]	<0.001	659
MDE_year	0 (0-1)	0 (0-1)	0.9 [0.7;1.1]	0.157	479	0 (0-1)	0 (0-1)	1.0 [0.8;1.2]	0.830	478
UP_year	0 (0-1)	0 (0-2)	1.0 [1.0;1.1]	0.608	528	0 (0-2)	0 (0-1)	0.9 [0.9;1.0]	0.474	527
sa_ever: 1	135 (41%)	70 (36%)	0.8 [0.5;1.1]	0.235	525	112 (40%)	93 (38%)	0.9 [0.6;1.3]	0.698	525
current_smoking: 1	91 (22%)	83 (32%)	1.7 [1.2;2.4]	0.004	670	96 (28%)	77 (23%)	0.8 [0.5;1.1]	0.181	669
Misuse_alcohol_lifetime: 1	59 (14%)	45 (18%)	1.3 [0.8;2.0]	0.265	662	56 (17%)	48 (15%)	0.9 [0.6;1.3]	0.597	661
Misuse_cannabis_lifetime: 1	44 (11%)	22 (9%)	0.8 [0.5;1.4]	0.512	664	24 (7%)	42 (13%)	1.9 [1.1;3.3]	0.019	663
Misuse_cocaine_lifetime: 1	5 (2%)	1 (0%)	0.4 [<0.1;2.4]	0.417	532	0 (0%)	6 (2%)	. [.;.]	0.010	531
Misuse_other_lifetime_bis: 1	18 (6%)	10 (5%)	0.9 [0.4;2.0]	1.000	523	10 (4%)	18 (7%)	2.1 [1.0;4.9]	0.090	522

	antidepressants					benzodiazepines				
	0	1	OR	p.overall	N	0	1	OR	p.overall	N
	N=380	*N=289*				*N=543*	*N=124*			
age	36 (27-46)	37 (28-48)	1.0 [1.0;1.0]	0.508	669	34 (27-46)	43 (33-51)	1.0 [1.0;1.0]	<0.001	667
gender_txt:				0.001	669				0.314	667
Men	174 (46%)	94 (33%)	Ref.			222 (41%)	44 (35%)	Ref.		
Women	206 (54%)	195 (67%)	1.7 [1.3;2.4]			321 (59%)	80 (65%)	1.3 [0.8;1.9]		
site:				0.883	669				<0.001	667
NW	240 (63%)	185 (64%)	Ref.			384 (71%)	41 (33%)	Ref.		
FR	140 (37%)	104 (36%)	1.0 [0.7;1.3]			159 (29%)	83 (67%)	4.9 [3.2;7.5]		
bipolar_type:				<0.001	669				0.254	667
1	307 (81%)	172 (60%)	Ref.			394 (73%)	83 (67%)	Ref.		
2	73 (19%)	117 (40%)	2.9 [2.0;4.1]			149 (27%)	41 (33%)	1.3 [0.9;2.0]		
bipolar_duration	12 (7-23)	13 (7-22)	1.0 [1.0;1.0]	0.547	527	12 (6-21)	17 (9-28)	1.0 [1.0;1.1]	<0.001	525
bipolar_AAO	22 (18-30)	20 (16-28)	1.0 [1.0;1.0]	0.034	527	21 (17-28)	21 (17-31)	1.0 [1.0;1.0]	0.677	525
Bipolar_any_psychotic: 1	256 (69%)	137 (48%)	0.4 [0.3;0.6]	<0.001	659	319 (60%)	72 (60%)	1.0 [0.7;1.5]	1.000	657
MDE_year	0 (0-1)	0 (0-1)	1.2 [1.0;1.5]	<0.001	478	0 (0-1)	0 (0-1)	0.7 [0.5;1.0]	0.562	476
UP_year	0 (0-1)	0 (0-2)	1.1 [1.0;1.2]	0.234	527	0 (0-1)	1 (0-2)	1.1 [1.0;1.2]	0.001	525
sa_ever: 1	99 (34%)	105 (44%)	1.5 [1.1;2.2]	0.028	524	146 (35%)	58 (53%)	2.0 [1.3;3.1]	0.001	523
current_smoking: 1	100 (26%)	74 (26%)	1.0 [0.7;1.4]	0.906	669	130 (24%)	42 (34%)	1.6 [1.1;2.5]	0.030	667
Misuse_alcohol_lifetime: 1	60 (16%)	44 (15%)	1.0 [0.6;1.5]	0.941	661	79 (15%)	25 (21%)	1.5 [0.9;2.5]	0.124	659
Misuse_cannabis_lifetime: 1	35 (9%)	31 (11%)	1.2 [0.7;2.0]	0.595	663	52 (10%)	13 (11%)	1.1 [0.6;2.1]	0.839	661
Misuse_cocaine_lifetime: 1	2 (1%)	4 (2%)	2.3 [0.4;19.1]	0.418	531	5 (1%)	1 (1%)	0.8 [<0.1;5.5]	1.000	529
Misuse_other_lifetime_bis: 1	12 (4%)	16 (7%)	1.6 [0.8;3.6]	0.277	522	24 (6%)	4 (4%)	0.6 [0.2;1.7]	0.544	520
