# Supplementary material for: Preventive Medication Patterns in Bipolar Disorder and Their Relationship With Comorbid Substance Use Disorders in a Cross-National Observational Study
Source: Front Psychiatry. 2022 May 3;13:813256. doi: 10.3389/fpsyt.2022.813256 (PMC9110763; doi:10.3389/fpsyt.2022.813256)
Supplement: Supplementary file 6 [file Table_3.pdf]

Descriptive statistics and nominal associations of medication variables

	compliance					lithium					antiepileptics					antipsychotics					antidepressants					benzodiazepines				
	compliant	partly_compliant	non_compliant	p.trend	N	0	1	OR	p.overall	N	0	1	OR	p.overall	N	0	1	OR	p.overall	N	0	1	OR	p.overall	N	0	1	OR	p.overall	N
	N=221	*N=168*	*N=36*			*N=328*	*N=97*				*N=286*	*N=139*				*N=171*	*N=254*				*N=240*	*N=185*				*N=384*	*N=41*			
age	31 (25-42)	33 (26-43)	30 (24-40)	0.622	425	31 (24-41)	35 (26-45)	1.0 [1.0;1.0]	0.051	425	32 (25-43)	31 (25-41)	1.0 [1.0;1.0]	0.957	425	31 (25-42)	32 (24-42)	1.0 [1.0;1.0]	0.695	425	31 (25-42)	32 (25-43)	1.0 [1.0;1.0]	0.494	425	31 (25-42)	36 (25-46)	1.0 [1.0;1.0]	0.479	425
gender_txt:				0.098	425				0.927	425				0.640	425				0.283	425				0.031	425				0.403	425
Men	93 (42%)	63 (38%)	10 (28%)			129 (39%)	37 (38%)	Ref.			109 (38%)	57 (41%)	Ref.			61 (36%)	105 (41%)	Ref.			105 (44%)	61 (33%)	Ref.			147 (38%)	19 (46%)	Ref.		
Women	128 (58%)	105 (62%)	26 (72%)			199 (61%)	60 (62%)	1.0 [0.7;1.7]			177 (62%)	82 (59%)	0.9 [0.6;1.3]			110 (64%)	149 (59%)	0.8 [0.5;1.2]			135 (56%)	124 (67%)	1.6 [1.1;2.4]			237 (62%)	22 (54%)	0.7 [0.4;1.4]		
site: NW	221 (100%)	168 (100%)	36 (100%)	.	425	328 (100%)	97 (100%)	Ref.	.	425	286 (100%)	139 (100%)	Ref.	.	425	171 (100%)	254 (100%)	Ref.	.	425	240 (100%)	185 (100%)	Ref.	.	425	384 (100%)	41 (100%)	Ref.	.	425
bipolar_type:				<0.001	425				<0.001	425				0.020	425				<0.001	425				0.001	425				1.000	425
1	171 (77%)	112 (67%)	13 (36%)			212 (65%)	84 (87%)	Ref.			210 (73%)	86 (62%)	Ref.			93 (54%)	203 (80%)	Ref.			184 (77%)	112 (61%)	Ref.			267 (70%)	29 (71%)	Ref.		
2	50 (23%)	56 (33%)	23 (64%)			116 (35%)	13 (13%)	0.3 [0.1;0.5]			76 (27%)	53 (38%)	1.7 [1.1;2.6]			78 (46%)	51 (20%)	0.3 [0.2;0.5]			56 (23%)	73 (39%)	2.1 [1.4;3.3]			117 (30%)	12 (29%)	1.0 [0.5;1.9]		
bipolar_duration	10 (5-16)	11 (5-19)	10 (6-17)	0.432	284	9 (5-17)	10 (6-20)	1.0 [1.0;1.0]	0.498	284	9 (5-19)	11 (6-16)	1.0 [1.0;1.0]	0.612	284	11 (6-19)	9 (5-18)	1.0 [1.0;1.0]	0.172	284	10 (5-18)	10 (5-19)	1.0 [1.0;1.0]	0.759	284	9 (5-17)	15 (8-22)	1.0 [1.0;1.1]	0.036	284
bipolar_AAO	22 (17-30)	19 (16-26)	18 (15-26)	0.016	284	20 (16-27)	22 (17-30)	1.0 [1.0;1.0]	0.508	284	20 (16-28)	20 (16-26)	1.0 [1.0;1.0]	0.761	284	20 (16-27)	20 (17-28)	1.0 [1.0;1.0]	0.596	284	22 (17-30)	19 (16-26)	1.0 [1.0;1.0]	0.023	284	20 (17-28)	18 (13-26)	1.0 [0.9;1.0]	0.110	284
Bipolar_any_psychotic: 1	142 (65%)	90 (54%)	14 (40%)	0.002	419	187 (58%)	59 (61%)	1.2 [0.7;1.9]	0.614	419	174 (62%)	72 (52%)	0.7 [0.4;1.0]	0.055	419	66 (39%)	180 (72%)	4.1 [2.7;6.2]	<0.001	419	154 (65%)	92 (50%)	0.5 [0.4;0.8]	0.003	419	220 (58%)	26 (65%)	1.3 [0.7;2.7]	0.496	419
MDE_year	0 (0-1)	0 (0-1)	0 (0-1)	0.022	271	0 (0-1)	0 (0-1)	1.1 [0.8;1.4]	0.479	271	0 (0-1)	0 (0-1)	0.9 [0.7;1.3]	0.981	271	0 (0-1)	0 (0-1)	0.8 [0.7;1.1]	0.051	271	0 (0-1)	0 (0-1)	1.2 [0.9;1.5]	0.024	271	0 (0-1)	0 (0-1)	0.9 [0.5;1.5]	0.866	271
UP_year	0 (0-1)	0 (0-1)	0 (0-1)	0.542	284	0 (0-1)	0 (0-1)	1.0 [0.9;1.1]	0.655	284	0 (0-1)	0 (0-1)	1.1 [1.0;1.2]	0.488	284	0 (0-1)	0 (0-1)	0.9 [0.8;1.0]	0.037	284	0 (0-1)	0 (0-1)	1.1 [1.0;1.2]	0.932	284	0 (0-1)	0 (0-2)	1.0 [0.8;1.2]	0.241	284
sa_ever: 1	35 (25%)	49 (39%)	8 (40%)	0.018	284	65 (30%)	27 (39%)	1.4 [0.8;2.5]	0.261	284	72 (36%)	20 (24%)	0.6 [0.3;1.0]	0.090	284	34 (30%)	58 (34%)	1.2 [0.7;2.0]	0.585	284	39 (26%)	53 (39%)	1.8 [1.1;3.0]	0.026	284	81 (32%)	11 (41%)	1.5 [0.6;3.4]	0.448	284
current_smoking: 1	40 (18%)	31 (18%)	4 (11%)	0.508	425	57 (17%)	18 (19%)	1.1 [0.6;1.9]	0.908	425	38 (13%)	37 (27%)	2.4 [1.4;3.9]	0.001	425	26 (15%)	49 (19%)	1.3 [0.8;2.3]	0.340	425	43 (18%)	32 (17%)	1.0 [0.6;1.6]	0.970	425	67 (17%)	8 (20%)	1.2 [0.5;2.5]	0.909	425
Misuse_alcohol_lifetime: 1	28 (13%)	24 (14%)	4 (11%)	0.933	425	47 (14%)	9 (9%)	0.6 [0.3;1.3]	0.262	425	36 (13%)	20 (14%)	1.2 [0.6;2.1]	0.717	425	26 (15%)	30 (12%)	0.7 [0.4;1.3]	0.385	425	30 (12%)	26 (14%)	1.1 [0.6;2.0]	0.745	425	52 (14%)	4 (10%)	0.7 [0.2;1.9]	0.661	425
Misuse_cannabis_lifetime: 1	18 (8%)	16 (10%)	5 (14%)	0.300	425	35 (11%)	4 (4%)	0.4 [0.1;1.0]	0.078	425	28 (10%)	11 (8%)	0.8 [0.4;1.6]	0.653	425	14 (8%)	25 (10%)	1.2 [0.6;2.5]	0.683	425	19 (8%)	20 (11%)	1.4 [0.7;2.8]	0.392	425	36 (9%)	3 (7%)	0.8 [0.2;2.4]	1.000	425
Misuse_cocaine_lifetime: 1	2 (1%)	2 (2%)	0 (0%)	0.781	287	4 (2%)	0 (0%)	. [.;.]	0.575	287	4 (2%)	0 (0%)	. [.;.]	0.327	287	0 (0%)	4 (2%)	. [.;.]	0.157	287	2 (1%)	2 (1%)	1.1 [0.1;10.6]	1.000	287	4 (2%)	0 (0%)	. [.;.]	1.000	287
Misuse_other_lifetime_bis: 1	7 (5%)	14 (11%)	1 (5%)	0.263	287	21 (10%)	1 (1%)	0.2 [<0.1;0.7]	0.043	287	14 (7%)	8 (10%)	1.5 [0.6;3.6]	0.578	287	8 (7%)	14 (8%)	1.1 [0.5;3.0]	0.941	287	8 (5%)	14 (10%)	2.0 [0.8;5.2]	0.183	287	20 (8%)	2 (7%)	1.0 [0.1;3.7]	1.000	287

File failed to load: /extensions/MathMenu.js
