# Supplementary material for: Preventive Medication Patterns in Bipolar Disorder and Their Relationship With Comorbid Substance Use Disorders in a Cross-National Observational Study
Source: Front Psychiatry. 2022 May 3;13:813256. doi: 10.3389/fpsyt.2022.813256 (PMC9110763; doi:10.3389/fpsyt.2022.813256)
Supplement: Supplementary file 7 [file Table_4.pdf]

Descriptive statistics and nominal associations of medication variables

| | compliance | | | | |
|---|---|---|---|---|---|
| | **compliant** | **partly_compliant** | **non_compliant** | **p.trend** | **N** |
| | *N=100* | *N=128* | *N=17* | | |
| age | 42 (13) | 44 (11) | 46 (13) | 0.186 | 245 |
| gender_txt: | | | | 0.014 | 245 |
| Men | 52 (52%) | 44 (34%) | 6 (35%) | | |
| Women | 48 (48%) | 84 (66%) | 11 (65%) | | |
| site: FR | 100 (100%) | 128 (100%) | 17 (100%) | . | 245 |
| bipolar_type: | | | | <0.001 | 245 |
| 1 | 91 (91%) | 81 (63%) | 12 (71%) | | |
| 2 | 9 (9%) | 47 (37%) | 5 (29%) | | |
| bipolar_duration | 16 (8-27) | 17 (11-27) | 26 (8-34) | 0.100 | 244 |
| bipolar_AAO | 22 (18-30) | 22 (18-30) | 20 (18-28) | 0.996 | 244 |
| Bipolar_any_psychotic: 1 | 76 (77%) | 64 (51%) | 8 (47%) | <0.001 | 241 |
| MDE_year | 0 (0-0) | 0 (0-0) | 0 (0-1) | 0.002 | 208 |
| UP_year | 0 (0-1) | 1 (0-3) | 2 (0-2) | 0.018 | 244 |
| sa_ever: 1 | 37 (38%) | 69 (55%) | 7 (41%) | 0.089 | 241 |
| current_smoking: 1 | 38 (38%) | 55 (43%) | 6 (35%) | 0.740 | 245 |
| Misuse_alcohol_lifetime: 1 | 18 (18%) | 26 (21%) | 4 (24%) | 0.523 | 237 |
| Misuse_cannabis_lifetime: 1 | 10 (10%) | 15 (12%) | 2 (12%) | 0.671 | 239 |
| Misuse_cocaine_lifetime: 1 | 0 (0%) | 2 (2%) | 0 (0%) | 0.425 | 245 |
| Misuse_other_lifetime_bis: 1 | 3 (3%) | 3 (2%) | 0 (0%) | 0.518 | 236 |

| | lithium | | | | |
|---|---|---|---|---|---|
| | **0** | **1** | **OR** | **p.overall** | **N** |
| | *N=137* | *N=99* | | | |
| age | 44 (13) | 43 (12) | 1.0 [1.0;1.0] | 0.579 | 236 |
| gender_txt: | | | | 0.905 | 236 |
| Men | 59 (43%) | 41 (41%) | Ref. | | |
| Women | 78 (57%) | 58 (59%) | 1.1 [0.6;1.8] | | |
| site: FR | 137 (100%) | 99 (100%) | Ref. | . | 236 |
| bipolar_type: | | | | 0.322 | 236 |
| 1 | 102 (74%) | 80 (81%) | Ref. | | |
| 2 | 35 (26%) | 19 (19%) | 0.7 [0.4;1.3] | | |
| bipolar_duration | 17 (9-28) | 16 (8-24) | 1.0 [1.0;1.0] | 0.322 | 235 |
| bipolar_AAO | 22 (18-28) | 22 (18-30) | 1.0 [1.0;1.0] | 0.606 | 235 |
| Bipolar_any_psychotic: 1 | 86 (63%) | 60 (63%) | 1.0 [0.6;1.8] | 1.000 | 232 |
| MDE_year | 0 (0-0) | 0 (0-0) | 1.1 [0.8;1.6] | 0.460 | 202 |
| UP_year | 0 (0-2) | 0 (0-2) | 1.0 [0.8;1.1] | 0.637 | 235 |
| sa_ever: 1 | 57 (42%) | 50 (52%) | 1.5 [0.9;2.5] | 0.203 | 232 |
| current_smoking: 1 | 52 (38%) | 43 (43%) | 1.3 [0.7;2.1] | 0.476 | 236 |
| Misuse_alcohol_lifetime: 1 | 31 (23%) | 16 (17%) | 0.7 [0.3;1.3] | 0.275 | 228 |
| Misuse_cannabis_lifetime: 1 | 15 (11%) | 12 (12%) | 1.1 [0.5;2.5] | 1.000 | 230 |
| Misuse_cocaine_lifetime: 1 | 0 (0%) | 2 (2%) | . [.;.] | 0.175 | 236 |
| Misuse_other_lifetime_bis: 1 | 1 (1%) | 5 (5%) | 6.5 [1.0;174.3] | 0.085 | 227 |

| | antiepileptics | | | | |
|---|---|---|---|---|---|
| | **0** | **1** | **OR** | **p.overall** | **N** |
| | *N=128* | *N=117* | | | |
| age | 43 (13) | 43 (12) | 1.0 [1.0;1.0] | 0.900 | 245 |
| gender_txt: | | | | 0.642 | 245 |
| Men | 51 (40%) | 51 (44%) | Ref. | | |
| Women | 77 (60%) | 66 (56%) | 0.9 [0.5;1.4] | | |
| site: FR | 128 (100%) | 117 (100%) | Ref. | . | 245 |
| bipolar_type: | | | | 1.000 | 245 |
| 1 | 96 (75%) | 88 (75%) | Ref. | | |
| 2 | 32 (25%) | 29 (25%) | 1.0 [0.6;1.8] | | |
| bipolar_duration | 16 (8-26) | 17 (9-27) | 1.0 [1.0;1.0] | 0.477 | 244 |
| bipolar_AAO | 22 (18-30) | 22 (18-30) | 1.0 [1.0;1.0] | 0.688 | 244 |
| Bipolar_any_psychotic: 1 | 74 (59%) | 74 (64%) | 1.2 [0.7;2.0] | 0.549 | 241 |
| MDE_year | 0 (0-0) | 0 (0-0) | 0.9 [0.6;1.3] | 0.452 | 208 |
| UP_year | 0 (0-2) | 0 (0-2) | 1.0 [0.9;1.1] | 0.464 | 244 |
| sa_ever: 1 | 63 (50%) | 50 (43%) | 0.8 [0.5;1.3] | 0.377 | 241 |
| current_smoking: 1 | 53 (41%) | 46 (39%) | 0.9 [0.5;1.5] | 0.839 | 245 |
| Misuse_alcohol_lifetime: 1 | 23 (18%) | 25 (22%) | 1.3 [0.7;2.4] | 0.556 | 237 |
| Misuse_cannabis_lifetime: 1 | 16 (13%) | 11 (10%) | 0.8 [0.3;1.7] | 0.637 | 239 |
| Misuse_cocaine_lifetime: 1 | 1 (1%) | 1 (1%) | 1.1 [<0.1;43.0] | 1.000 | 245 |
| Misuse_other_lifetime_bis: 1 | 4 (3%) | 2 (2%) | 0.6 [0.1;3.1] | 0.685 | 236 |

| | antipsychotics | | | | |
|---|---|---|---|---|---|
| | **0** | **1** | **OR** | **p.overall** | **N** |
| | *N=169* | *N=75* | | | |
| age | 44 (12) | 43 (13) | 1.0 [1.0;1.0] | 0.618 | 244 |
| gender_txt: | | | | 1.000 | 244 |
| Men | 70 (41%) | 31 (41%) | Ref. | | |
| Women | 99 (59%) | 44 (59%) | 1.0 [0.6;1.8] | | |
| site: FR | 169 (100%) | 75 (100%) | Ref. | . | 244 |
| bipolar_type: | | | | 0.045 | 244 |
| 1 | 120 (71%) | 63 (84%) | Ref. | | |
| 2 | 49 (29%) | 12 (16%) | 0.5 [0.2;0.9] | | |
| bipolar_duration | 17 (8-28) | 15 (10-23) | 1.0 [1.0;1.0] | 0.725 | 243 |
| bipolar_AAO | 22 (18-30) | 22 (18-30) | 1.0 [1.0;1.0] | 0.792 | 243 |
| Bipolar_any_psychotic: 1 | 91 (54%) | 56 (77%) | 2.7 [1.5;5.2] | 0.002 | 240 |
| MDE_year | 0 (0-0) | 0 (0-0) | 1.0 [0.7;1.4] | 0.555 | 207 |
| UP_year | 0 (0-2) | 1 (0-3) | 1.1 [1.0;1.2] | 0.015 | 243 |
| sa_ever: 1 | 78 (47%) | 35 (47%) | 1.0 [0.6;1.8] | 1.000 | 241 |
| current_smoking: 1 | 70 (41%) | 28 (37%) | 0.8 [0.5;1.5] | 0.646 | 244 |
| Misuse_alcohol_lifetime: 1 | 30 (18%) | 18 (26%) | 1.6 [0.8;3.0] | 0.248 | 236 |
| Misuse_cannabis_lifetime: 1 | 10 (6%) | 17 (24%) | 4.8 [2.1;11.5] | <0.001 | 238 |
| Misuse_cocaine_lifetime: 1 | 0 (0%) | 2 (3%) | . [.;.] | 0.094 | 244 |
| Misuse_other_lifetime_bis: 1 | 2 (1%) | 4 (6%) | 4.6 [0.8;38.2] | 0.070 | 235 |

| | antidepressants | | | | |
|---|---|---|---|---|---|
| | **0** | **1** | **OR** | **p.overall** | **N** |
| | *N=140* | *N=104* | | | |
| age | 43 (13) | 43 (12) | 1.0 [1.0;1.0] | 0.772 | 244 |
| gender_txt: | | | | 0.009 | 244 |
| Men | 69 (49%) | 33 (32%) | Ref. | | |
| Women | 71 (51%) | 71 (68%) | 2.1 [1.2;3.6] | | |
| site: FR | 140 (100%) | 104 (100%) | Ref. | . | 244 |
| bipolar_type: | | | | <0.001 | 244 |
| 1 | 123 (88%) | 60 (58%) | Ref. | | |
| 2 | 17 (12%) | 44 (42%) | 5.2 [2.8;10.2] | | |
| bipolar_duration | 17 (8-26) | 16 (10-29) | 1.0 [1.0;1.0] | 0.369 | 243 |
| bipolar_AAO | 22 (18-30) | 22 (18-29) | 1.0 [1.0;1.0] | 0.639 | 243 |
| Bipolar_any_psychotic: 1 | 102 (74%) | 45 (44%) | 0.3 [0.2;0.5] | <0.001 | 240 |
| MDE_year | 0 (0-0) | 0 (0-0) | 1.2 [0.9;1.8] | 0.001 | 207 |
| UP_year | 0 (0-2) | 1 (0-2) | 1.2 [1.0;1.3] | 0.048 | 243 |
| sa_ever: 1 | 60 (43%) | 52 (51%) | 1.3 [0.8;2.3] | 0.307 | 240 |
| current_smoking: 1 | 57 (41%) | 42 (40%) | 1.0 [0.6;1.7] | 1.000 | 244 |
| Misuse_alcohol_lifetime: 1 | 30 (22%) | 18 (18%) | 0.8 [0.4;1.5] | 0.547 | 236 |
| Misuse_cannabis_lifetime: 1 | 16 (12%) | 11 (11%) | 0.9 [0.4;2.1] | 1.000 | 238 |
| Misuse_cocaine_lifetime: 1 | 0 (0%) | 2 (2%) | . [.;.] | 0.181 | 244 |
| Misuse_other_lifetime_bis: 1 | 4 (3%) | 2 (2%) | 0.7 [0.1;3.8] | 1.000 | 235 |

| | benzodiazepines | | | | |
|---|---|---|---|---|---|
| | **0** | **1** | **OR** | **p.overall** | **N** |
| | *N=159* | *N=83* | | | |
| age | 42 (13) | 45 (12) | 1.0 [1.0;1.0] | 0.093 | 242 |
| gender_txt: | | | | 0.016 | 242 |
| Men | 75 (47%) | 25 (30%) | Ref. | | |
| Women | 84 (53%) | 58 (70%) | 2.1 [1.2;3.7] | | |
| site: FR | 159 (100%) | 83 (100%) | Ref. | . | 242 |
| bipolar_type: | | | | 0.018 | 242 |
| 1 | 127 (80%) | 54 (65%) | Ref. | | |
| 2 | 32 (20%) | 29 (35%) | 2.1 [1.2;3.9] | | |
| bipolar_duration | 17 (8-27) | 17 (10-29) | 1.0 [1.0;1.0] | 0.243 | 241 |
| bipolar_AAO | 22 (18-29) | 22 (18-31) | 1.0 [1.0;1.0] | 0.575 | 241 |
| Bipolar_any_psychotic: 1 | 99 (63%) | 46 (57%) | 0.8 [0.4;1.3] | 0.424 | 238 |
| MDE_year | 0 (0-0) | 0 (0-0) | 0.8 [0.5;1.2] | 0.266 | 205 |
| UP_year | 0 (0-2) | 1 (0-3) | 1.2 [1.0;1.3] | 0.022 | 241 |
| sa_ever: 1 | 65 (42%) | 47 (57%) | 1.8 [1.1;3.1] | 0.038 | 239 |
| current_smoking: 1 | 63 (40%) | 34 (41%) | 1.1 [0.6;1.8] | 0.949 | 242 |
| Misuse_alcohol_lifetime: 1 | 27 (17%) | 21 (27%) | 1.7 [0.9;3.3] | 0.141 | 234 |
| Misuse_cannabis_lifetime: 1 | 16 (10%) | 10 (12%) | 1.3 [0.5;2.9] | 0.763 | 236 |
| Misuse_cocaine_lifetime: 1 | 1 (1%) | 1 (1%) | 1.9 [<0.1;75.5] | 1.000 | 242 |
| Misuse_other_lifetime_bis: 1 | 4 (3%) | 2 (3%) | 1.0 [0.1;5.6] | 1.000 | 233 |
